# Supplementary material for: WordCommentsAnalyzer: A windows software tool for qualitative research
Source: F1000Res. 2018 Sep 4;7:536. Originally published 2018 May 3. [Version 2] doi: 10.12688/f1000research.14819.2 (PMC6137411; doi:10.12688/f1000research.14819.2)
Supplement: Supplementary file 3 [file f1000research-7-17614-s0002.tgz › b2a79496-4ada-47c4-9682-a99ce867836d.zip!/Eight Iranian Health Journals/Journal of Education and Community Health_2.docx]

© Journal of Education and Community Health. This work is licensed under CC BY-NC 4.0 (https://creativecommons.org/licenses/by-nc/4.0/).

Volume & Issue: دوره 4 - شماره 3

Title: تأثیر برنامه آموزشی تغذیه ای پیشگیری کننده از مسمومیت بارداری بر رفتارهای تغذیه ای زنان باردار

http://jech.umsha.ac.ir/article-1-345-fa.html

سابقه و هدف: مسمومیت حاملگی شایع‌ترین عارضه دوران بارداری بوده و یکی از سه علت اصلی مرگ زنان باردار می‌باشد. با توجه به شیوع بالای مسمومیت حاملگی در شهرستان پلدختر، این مطالعه با هدف بررسی تأثیر برنامه آموزشی تغذیه‌ای پیشگیری‌کننده از مسمومیت بارداری بر رفتارهای تغذیه‌ای زنان باردار انجام شد. مواد و روش‌‌ها: پژوهش حاضر یک مطالعه نیمه‌تجربی بود که در سال 1396 و با شرکت 120 زن باردار سه ماهه سوم مراجعه‌کننده به مراکز بهداشتی- درمانی شهرستان پلدختر انجام گرفت. شرکت‌کنندگان در پژوهش به روش نمونه‌گیری خوشه‌ای انتخاب شدند و به‌صورت تصادفی به دو گروه آزمون (60 نفر) و کنترل (60 نفر) تقسیم گردیدند. ابزار جمع‌آوری اطلاعات شامل: پرسش‌نامه محقق‌ساخته مشتمل بر اطلاعات جمعیت‌شناختی، سؤالات آگاهی، نگرش و عملکرد بود. داده‌ها در نرم‌افزار ‌SPSS 16  و با استفاده از آزمون‌های کای دو، دقیق فیشر، تی مستقل و تی زوجی تحلیل شدند. یافته‌ها: نتایج حاصل از این پژوهش تفاوت معناداری را در زمینه آگاهی، نگرش و عملکرد پس از مداخله بین گروه آزمون و کنترل نشان داد (P<0.001). همچنین تفاوت آماری معناداری بین میانگین نمرات آگاهی، نگرش و عملکرد قبل و بعد از مداخله در گروه آزمون وجود داشت (P<0.001). نتیجه‌گیری: نتایج پژوهش حاضر نشان داد که مداخله آموزشی در بهبود رفتارهای تغذیه‌ای پیشگیری‌کننده از مسمومیت حاملگی مؤثر می‌باشد؛ از این رو پیشنهاد می‌شود ضمن انجام مطالعات بیشتر برای تأیید نتایج، از این مداخله برای آموزش زنان باردار استفاده گردد.

Title: پیش بینی عوامل موثر بر رعایت احتیاطات استاندارد کنترل عفونت کارکنان اورژانس پیش بیمارستانی همدان بر اساس مدل اعتقاد بهداشتی

http://jech.umsha.ac.ir/article-1-372-fa.html

سابقه و هدف: احتیاطات استاندارد، راهبردی اساسی برای پیشگیری از مواجهات شغلی در کارکنان اورژانس پیش‌بیمارستانی می‌باشد؛ از این رو، این پژوهش با هدف تعیین عوامل پیش‌بینی‌کننده رعایت احتیاطات استاندارد کنترل عفونت بر‌اساس مدل اعتقاد بهداشتی انجام شد. مواد و روش‌‌ها: در این مطالعه توصیفی- تحلیلی، 84 نفر از کارکنان اورژانس پیش‌بیمارستانی شهر همدان در سال 1396 به روش سرشماری مورد بررسی قرار گرفتند. ابزار گردآوری داده‌ها پرسشنامه چندبخشی شامل: اطلاعات دموگرافیک، آگاهی، سازه‌های مدل اعتقاد بهداشتی و عملکرد بود که به‌صورت خود‌گزارشی تکمیل گردید. داده‌ها با استفاده از نرم‌افزار SPSS 21 توسط آزمون‌های آماری تی مستقل، همبستگی Pearson و رگرسیون خطی مورد تجزیه و تحلیل قرار گرفتند. یافته‌ها: میانگین سنی افراد مورد مطالعه 7.63±31.64 سال بود. همچنین، میانگین نمره آگاهی شرکت‌کنندگان در پژوهش در مورد احتیاطات استاندارد (15.13±46.85) در حد ضعیف و میانگین نمره عملکرد نمونه‌ها در حد متوسط (12.73±55.16) ارزیابی گردید. از سوی دیگر بین سازه‌های منافع درک‌شده، راهنمای عمل، حساسیت درک‌شده، خودکارآمدی درک‌شده و عملکرد ارتباط معناداری مشاهده شد (0.05>P). بین سازه منافع درک‌شده و آگاهی نیز ارتباط معناداری وجود داشت (0.009=P)؛ اما نتایج آنالیز رگرسیون خطی معنادار نبود (0.05 نتیجه‌گیری: بر‌اساس نتایج این پژوهش با تقویت سازه‌های منافع درک‌شده، حساسیت درک‌شده، خودکارآمدی درک‌شده و راهنمای عمل می‌توان بهبود رعایت احتیاطات استاندارد کنترل عفونت را در کارکنان اورژانس پیش‌بیمارستانی انتظار داشت.

Title: رابطه انزوای اجتماعی و سلامت در سالمندان تهرانی

http://jech.umsha.ac.ir/article-1-369-fa.html

سابقه و هدف: با توجه به روند رو به رشد جمعیت سالمندان و نیز افزایش ناتوانایی‌های دوره سالمندی به دلیل بالا‌رفتن سن، توجه به انزوای اجتماعی سالمندان و نقش سلامت در کاهش این پدیده از اهمیت خاصی برخوردار می‌باشد. در این راستا هدف اصلی پژوهش حاضر، بررسی رابطه انزوای اجتماعی و سلامت در سالمندان شهر تهران است. مواد و روش‌‌ها: این مطالعه مقطعی- همبستگی در سال 1396 در ارتباط با 382 نفر از سالمندان 65 سال و بالاتر شهر تهران که با روش نمونه‌گیری خوشه‌ای چندمرحله‌ای انتخاب ‌شده بودند، انجام گرفت. ابزار گردآوری اطلاعات شامل: پرسشنامه سلامت عمومی Goldberg (GHQ: General Health Questionnaire)، پرسشنامه توانایی انجام فعالیت‌های روزانه زندگی (ADL: Activities of Daily Living) و پرسشنامه سلامت اجتماعی Keyes بود. تحلیل داده‌ها با استفاده از نرم‌افزار آماری SPSS 22 در 2 سطح توصیفی و تحلیلی از طریق آزمون‌ ضریب همبستگی Pearson صورت گرفت. یافته‌ها: 52.4 درصد از پاسخگویان دارای سلامت جسمانی، 56.8 درصد دارای سلامت اجتماعی و 60.5 درصد دارای سلامت روانی بودند و میانگین انزوای اجتماعی در سالمندان مورد مطالعه حدود 62 درصد بود. همچنین نتایج ضریب همبستگی Pearson نشان داد که بین سلامت روانی و اجتماعی سالمندان و انزوای اجتماعی آن‌ها رابطه معنا‌دار و معکوسی وجود دارد؛ به عبارت دیگر با افزایش سلامت روانی و اجتماعی، انزوای اجتماعی سالمندان کاهش می‌یابد. نتیجه‌گیری: بین سلامت و انزوای اجتماعی در سالمندان رابطه‌ای معکوس وجود دارد و برخورداری از سلامت روانی و اجتماعی می‌تواند بر کاهش انزوای اجتماعی این قشر آسیب‌پذیر اثر مثبتی داشته باشد.

Title: اثربخشی زوج‌درمانی هیجان مدار بر تعهد زناشویی و دلزدگی زناشویی در زوجین نابارور

http://jech.umsha.ac.ir/article-1-307-fa.html

سابقه و هدف: ناباروری در سراسر جهان و در همه‌ فرهنگ‌ها به‌عنوان یک تجربه استرس‌زا، بحرانی و تهدیدکننده‌ ثبات فردی، زناشویی، خانوادگی و اجتماعی شناخته شده است. در این راستا پژوهش حاضر با هدف تعیین اثربخشی زوج‌درمانی هیجان‌مدار بر تعهد زناشویی و دلزدگی زناشویی در زوجین نابارور انجام شد. مواد و روش‌‌ها: پژوهش حاضر یک مطالعه نیمه‌تجربی با طرح پیش‌آزمون-پس‌آزمون همراه با گروه کنترل بود. جامعه آماری را زوج‌های نابارور مراجعه‌کننده به مراکز خصوصی زنان و نازایی شهر قم در نیمه‌ دوم سال ۱۳۹۴ و نیمه اول سال ۱۳۹۵ تشکیل ‌دادند. از میان جامعه آماری، ۳۰ زوج به‌صورت نمونه‌گیری هدفمند برگزیده شدند و به‌صورت تصادفی در 2 گروه آزمون (۱۵ زوج) و کنترل (۱۵ زوج) جای گرفتند و مطابق با طرح، تحت سنجش دوگانه پیش‌آزمون- پس‌آزمون (پس از ۳۵ روز) درآمدند. ابزار گردآوری اطلاعات برای این پژوهش، سیاهه تعهد زناشویی (DCI: Dimensions of Commitment Inventory) و مقیاس دلزدگی زناشویی (CBM: Couple Burnout Measure) بود. زوج‌های گروه آزمون به مدت ۱۰ جلسه (هفته‌ای 2 جلسه) تحت زوج‌درمانی هیجان‌مدار قرار گرفتند. برای تحلیل داده‌ها از نرم‌افزار SPSS و آزمون‌های تحلیل کوواریانس چند‌متغیری و تک‌متغیری استفاده شد. یافته‌ها: یافته‌های تحلیل کوواریانس چندمتغیری نشان داد که زوج‌درمانی هیجان‌مدار باعث افزایش معنادار میانگین نمرات پس‌آزمون متغیر تعهد زناشویی و خرده‌مقیاس‌های آن (تعهد شخصی، تعهد اخلاقی و تعهد ساختاری) در گروه آزمون گردید (P<0.05). همچنین نتایج تحلیل کوواریانس تک‌متغیری بیانگر آن بود که میانگین نمرات پس‌آزمون متغیر دلزدگی زناشویی در گروه آزمون و کنترل تفاوت معناداری با یکدیگر داشتند (P<0.01). نتیجه‌گیری: بر مبنای نتایج می‌توان گفت که آموزش مبتنی بر زوج‌درمانی هیجان‌مدار باعث بهبود تعهد زناشویی در زوجین گردیده و دلزدگی زناشویی‌ را کاهش می‌دهد.

Title: مقایسه سبک زندگی ارتقادهنده سلامت در افراد مبتلا و غیرمبتلا به HIV شهر تهران

http://jech.umsha.ac.ir/article-1-276-fa.html

سابقه و هدف: سبک زندگی یکی از مسائلی است که می‌تواند در ابتلا به بیماری‌ها و درمان آن‌ها نقش مهمی داشته باشد. در این راستا، هدف از پژوهش حاضر مقایسه سبک زندگی ارتقا‌دهنده سلامت در افراد مبتلا و غیرمبتلا به HIV شهر تهران بود. مواد و روش‌‌ها: پژوهش مقطعی حاضر در سال 95-1393 در ارتباط با 147 فرد مبتلا به HIV که برای درمان به بیمارستان امام خمینی (ره) شهر تهران مراجعه کرده بودند و نیز 150 فرد غیر‌مبتلا در شهر تهران انجام شد. در این پژوهش پرسش‌نامه سبک زندگی ارتقا‌دهنده سلامت (HPLP-II: Health-promoting Lifestyle Profile II) که شامل 6 بعد مسئولیت‌پذیری در مورد سلامت، تغذیه، فعالیت بدنی، مدیریت استرس، روابط بین فردی و رشد معنوی می‌باشد مورد استفاده قرار گرفت. علاوه‌براین، نمونه‌گیری با استفاده از روش نمونه‌گیری در دسترس انجام گرفت و برای تحلیل داده‌ها از آزمون تحلیل واریانس چند‌متغیره و نرم‌افزار SPSS 22 بهره گرفته شد. یافته‌ها: نتایج آزمون تحلیل واریانس چند‌متغیری نشان داد که بین دو گروه در متغیر سبک زندگی تفاوت معناداری وجود دارد (P<0.001). بدین‌ترتیب که میزان مسئولیت‌پذیری سلامت (P<0.001، 44/42=(1،245)F) و فعالیت جسمانی (P<0.05، 03/6=(1،245)F) در گروه مبتلا به HIV کمتر از گروه غیرمبتلا به HIV بود؛ اما در مؤلفه‌های تغذیه، روابط بین فردی، رشد معنوی و مدیریت استرس تفاوت معناداری بین دو گروه مشاهده نشد (P>0.05). نتیجه‌گیری: با توجه به نبود اطلاعات در مورد سبک زندگی افراد مبتلا به HIV در ایران، آگاهی از سبک زندگی این افراد در جنبه‌های مختلف می‌تواند اطلاعات ارزشمندی را برای ارتقای وضعیت زندگی و برنامه‌های مداخلات درمانی به‌دست دهد. در این ارتباط، پژوهش‌های آینده باید بر ارتقای سبک زندگی در میان افراد مبتلا به HIV تمرکز بیشتری داشته باشند.

Title: رابطه شفقت خود با سواد سلامت دانش‌آموزان دوره اول متوسطه‌‌ در شهر گناباد

http://jech.umsha.ac.ir/article-1-317-fa.html

سابقه و هدف: افرادی‌ که از تاثیرات مثبت مهربانی آگاهند، از آن بعنوان وسیله‌ای برای حفظ و کنترل سلامتی خود استفاده می‌کنند. هدف از انجام این پژوهش بررسی رابطه‌ی شفقت خود بر سواد سلامت دانش‌آموزان متوسطه‌ اول شهر گناباد بوده است که در سال تحصیلی 96-95 صورت گرفت. مواد و روش‌ها: مطالعه حاضر از نوع توصیفی همبستگی بود. جامعه‌ی آماری شامل تمامی دانش‌آموزان دختر و پسر متوسطه اول شهرگناباد که مجموع آنها 2725 نفر بوده و بر اساس شیوه نمونه گیری تصادفی طبقه ای با توجه به جدول مورگان نمونه‌ای 351 نفری درنظرگرفته شد. برای گردآوری اطلاعات از پرسش نامه شفقت خود و پرسش نامه سواد سلامت استفاده شد. برای تحلیل داده‌‌ها از آزمون‌‌های آماری ضریب همبستگی پیرسون، آزمون رگرسیون چندگانه، آزمون تی مستقل و آزمون آنووا با استفاده از نرم افزار SPSS v.20 استفاده شد. یافته‌ها:میانگین و انحراف استاندارد نمره کل سواد سلامت 17.41±77.11 و نمره کل شفقت خود 12.9±71.6 بود. نتایج پژوهش حاضر نشان داد که بین سواد سلامت و شفقت خود دانش آموزان رابطه مثبت و معناداری وجود دارد (0.311=r). بین میزان سواد سلامت دانش آموزان دختر و پسر تفاوت وجود دارد (0.011=P). بین میزان شفقت خود دانش آموزان دختر و پسر تفاوت وجود ندارد (0.109=P)، بین میزان سواد سلامت دانش آموزان با توجه به پایه تحصیلی تفاوت وجود ندارد (0.051=P). نتیجه گیری: این پژوهش نشان داد که رابطه معناداری بین شفقت خود و سواد سلامت وجود دارد. بنابراین می توان با برگزاری دوره های آموزشی، کارگاه های آموزشی، پخش برنامه‌های صوتی و تصویری، توزیع تراکت‌های آموزشی در این زمینه فرهنگ‌سازی شود تا سواد سلامت دانش آموزان افزایش یافته و از تحمیل هزینه‌های سنگین بعدی ممانعت شود.

Title: عوامل مرتبط با قصد انجام فعالیت جسمانی بر اساس نظریه رفتار برنامه‌ریزی شده در دانش آموزان دختر دبیرستانی شهر اهواز

http://jech.umsha.ac.ir/article-1-357-fa.html

سابقه و هدف: فعالیت جسمانی از جنبه‌های مهم ارتقای سلامت و یکی از پیش‌بینی‌کننده‌های مرگ و ناتوانی در سراسر جهان است؛ از این رو پژوهش حاضر با هدف بررسی عوامل مرتبط با قصد انجام فعالیت جسمانی بر‌اساس نظریه رفتار برنامه‌ریزی‌شده در دانش‌آموزان دختر دبیرستانی شهر اهواز انجام شد. مواد و روش‌‌ها: مطالعه مقطعی- توصیفی- تحلیلی حاضر در سال 1395 بر روی 163 دانش‌آموز دختر دبیرستانی که به‌طور تصادفی ساده از 2 مدرسه دخترانه دولتی شهر اهواز انتخاب شده بودند، انجام شد. دانش‌آموزان پرسشنامه اطلاعات دموگرافیک و نظریه رفتار برنامه‌ریزی‌شده شامل: سازه نگرش، نرم‌های انتزاعی، کنترل رفتاری درک‌شده و قصد را به روش خودگزارش‌دهی تکمیل کردند. اطلاعات حاصل توسط نرم‌افزار SPSS 23 و با استفاده از آزمون‌های ضریب همبستگی Pearson و رگرسیون خطی تجزیه و تحلیل گردیدند. یافته‌ها: میانگین سنی شرکت‌کنندگان در پژوهش 0.89±‌13.76 سال بود. در تحلیل رگرسیونی، نگرش و کنترل رفتاری درک‌شده پیش‌بینی‌کننده قصد انجام فعالیت جسمانی بودند. این مدل توانست 40 درصد از واریانس قصد رفتاری را پیش‌بینی کند. شایان ذکر است که نرم‌های انتزاعی پیش‌بینی‌کننده معناداری برای قصد نبودند. نتیجه‌گیری: مطابق با یافته‌ها، استفاده از نظریه رفتار برنامه‌ریزی‌شده و توجه بیشتر به سازه نگرش به‌عنوان قوی‌ترین فاکتور پیش‌بینی‌کننده قصد در طراحی مداخلات آموزشی جهت ارتقای فعالیت جسمانی در بین دانش‌آموزان دختر، نویدبخش اثرات سودمندی خواهد بود.
